# Cell-Mediated Immune Responses to COVID-19 Infection

**DOI:** 10.3389/fimmu.2020.01662

**Published:** 2020-07-03

**Authors:** Amélie Guihot, Elena Litvinova, Brigitte Autran, Patrice Debré, Vincent Vieillard

**Affiliations:** ^1^Sorbonne Université, Inserm U1135, CNRS ERL 8255, Centre d'Immunologie et des Maladies Infectieuses (CIMI-Paris), Paris, France; ^2^Assistance Publique-Hôpitaux de Paris (AP-HP), Département d'Immunologie, Groupe Hospitalier Pitié-Salpêtrière, Paris, France

**Keywords:** SARS - CoV-2, COVID-19, innate immunity, adaptive immunity, coronavirus

## Abstract

An unprecedented outbreak of pneumonia caused by a novel coronavirus (CoV), subsequently termed COVID-19 by the World Health Organization, emerged in Wuhan City (China) in December 2019. Despite rigorous containment and quarantine efforts, the incidence of COVID-19 continues to expand, causing explosive outbreaks in more than 160 countries with waves of morbidity and fatality, leading to significant public health problems. In the past 20 years, two additional epidemics caused by CoVs have occurred: severe acute respiratory syndrome-CoV, which has caused a large-scale epidemic in China and 24 other countries; and respiratory syndrome-CoV of the Middle East in Saudi Arabia, which continues to cause sporadic cases. All of these viruses affect the lower respiratory tract and manifest as pneumonia in humans, but the novel SARS-Cov-2 appears to be more contagious and has spread more rapidly worldwide. This mini-review focuses on the cellular immune response to COVID-19 in human subjects, compared to other clinically relevant coronaviruses to evaluate its role in the control of infection and pathogenesis and accelerate the development of a preventive vaccine or immune therapies.

## Introduction

On December 31, 2019, a cluster of atypical pneumonia was reported in the Chinese city of Wuhan, mediated by a novel coronavirus (CoV) called SARS-CoV-2 (1, 2). The outbreak of this “coronavirus disease 2019” (COVID-19) has been declared a global pandemic by the World Health Organization (WHO), with more than 7 million cases in early June 2020 (3, 4) with a case-fatality rate of about 1%, as well as significant economic and social consequences. To date, no approved antiviral agents or efficient vaccines are available against the SARS-COV-2. For these reasons, necessary public health measures have been deployed, including worldwide quarantining of the populations and the use of barrier gestures to stop the progression of the SARS-COV-2.

CoVs are a class of positive-sense single-stranded RNA viruses found in a wide range of host species, including birds and mammals. Many of beta-CoV cause intestinal and respiratory infections in animals and humans. The zoonotic source of COVID-19 is not confirmed; however, sequencing of the SARS-CoV-2 reveals up to 80% identity with SARS-CoV and even more with several bat CoVs (5). This similarity suggests that bats could be the key reservoir, from which the virus was possibly directly transmitted to humans or through another unknown intermediate host. A phylogenetic analysis of 160 genomes of patients with COVID-19 revealed three major variants, named A, B, and C; the A-type being the ancestral type, firstly detected in China. The A and C types are found in significant proportions in Europe and America, whereas the B type is the most common in East Asia (6).

In 2002–2003, a first “atypical pneumonia,” called severe acute respiratory syndrome (SARS) was reported in Guangdong Province in China. The disease then spread to 37 countries to cause more than 8,000 cases, with a case-fatality rate of ~10% (7). At that time, SARS had already posed a worldwide public health threat, with a major impact on the economy. More recently, the Middle East respiratory syndrome (MERS) spread to 27 countries, causing around 2,500 cases. Among the CoVs, MERS has the highest case fatality rate (about 30%), but it is rarely transmitted between humans, only via camel (8). Thus, for the third time in a few decades, a new CoV has crossed species to infect human populations. However, compared with the other two CoVs, SARS-CoV-2 is much more contagious. Until now, more than 7 million cases have been diagnosed globally, with over 400,000 fatalities worldwide through early June 2020, with a basic reproductive number estimated to be from 2.2 to 3.3 and a mortality rate of around 2.3% (3, 9).

Like the other CoVs, SARS-CoV-2 possesses a typical envelope structure with spike proteins at the surface; this characteristic certainly plays a major role in interspecies transmission. Based on similarities in spike structure characteristics between SARS-CoV-2 and SARS-CoV, several research groups have demonstrated that SARS-CoV-2 also utilizes the human angiotensin-converting enzyme 2 (ACE2) receptor as a cellular entry receptor (10, 11). ACE2 is mainly expressed in vascular endothelial cells and the renal tubular epithelium. PCR analysis revealed that ACE2 is also expressed in the lungs and gastrointestinal tract, which are tissues shown to harbor viruses (12). It was also suggested that CD147 (basigin or the EMMPRIN protein) could be another cell-surface receptor for SARS-CoV-2 (13). By co-immunoprecipitation, ELISA, and immuno-electron microscopy, they show that anti-CD147 antibody (Meplazumab) could competitively inhibit the binding of spike protein (SP) with CD147 and thus prevent infection of target cells. A phase II clinical trial entitled “Clinical study of anti-CD147 humanized Meplazumab for injection to treat with 2019-nCoV pneumonia” (ClinicalTrials.gov identifier: NCT04275245) is currently underway in China aiming to prevent SARS-CoV-2 SP binding and subsequent infection (14). CD209L (L-SGN) has been identified as another possible alternative receptor for SARS-CoV-2, as previously described for the SARS-CoV virus (15).

This review highlights some of the most recent advances in our understanding of the role of innate and adaptive cellular immunity in COVID-19 infection and discusses potential links to pathogenesis.

## Immunopathology of COVID-19

### What of the Acute Infection?

The first symptoms associated with COVID-19 are mainly those of respiratory disease, although neurologic and digestive symptoms can also be observed. The primary mode of infection is human-to-human transmission through close contact, via the spraying of droplets from infected individuals, primarily through the nasal and larynx mucosa, followed by entrance into the lungs through the respiratory tract. Next, in more severe cases, damage/oedema due to extracellular fluid may let the virus enter the peripheral blood from the lungs, causing viremia. COVID-19 has a probable asymptomatic incubation period between 2 and 14 days during which the virus can be transmitted (16), but importantly, the duration of SARS-CoV-2 RNA detection has not been well-characterized. Zhou et al. (12) found that viral titers in nasopharyngeal aspirates diminish 10–15 days after the onset of symptoms, but remains high when the clinical disease worsens. It is, however, noteworthy that the presence of viral RNA in specimens does not always correlate with viral transmissibility; a major limitation remains the inability to differentiate between infective and non-infective (dead or antibody-neutralized) viruses. For SARS and MERS, it had previously been shown that viral RNA persisted in the respiratory tract for at least 3 weeks after disease onset in a majority of patients (17).

### What of the Severe Forms?

More than 80% of COVID-19 cases were asymptomatic or presented with mild symptoms, while the remaining cases were severe or critical (2, 18). It seems that the case-fatality rate of COVID-19 (about 1%) is lower than those of SARS (10%) and MERS (30%). Like other pathogenic CoVs, COVID-19 is associated with a typical influenza-like syndrome with fever, cough, fatigue and/or myalgia. Although diarrhea was reported in a foursome of patients with SARS and MERS, intestinal symptoms were rarely observed in patients with COVID-19 (2, 18, 19).

An early report in China found that 14% of COVID-19 patients were hospitalized, including 5% with ICU intervention (20). Similar proportions were observed later in Europe and the US (4). Among those who are seriously ill, acute hypoxemic respiratory failure due to acute respiratory distress syndrome (ARDS) is mainly observed (20, 21). At this stage, the need for mechanical ventilation is high, ranging from 40 to 100% (22); however, encephalitis and antiphospholipid syndrome are rare (23). Common complications of COVID-19 include acute kidney injury, elevated liver enzymes, and cardiac injury (23). The limited COVID-19 post mortem data show prominent alveolar edema, fibrin deposition, immune cell infiltration, and severe multi-organ damage, including renal, cardiac, and liver dysfunction (12, 24).

It was also reported that about 90% of COVID-19 hospitalized patients had at least one risk factor (www.cdc.gov/coronavirus/2019-ncov/index.html). Older age, in particular, as well as a higher sequential organ failure assessment (SOFA) score on admission, are associated with a higher probability of in-hospital death, whereas elevated levels of blood IL-6, high-sensitivity cardiac troponin I, and lymphopenia are more commonly seen in severe COVID-19 illness (12). It is still unknown why the cytokine storm may account for the severity of infection in elderly and immunocompromised (i.e., diabetics) but not in the young population who are mostly asymptomatic but have a fully functional immune system. However, the variability of clinical cases observed during exposure and infection with SARS-CoV-2 likely suggests that human genetic factors can also influence the response to this virus. However, to date, very few studies have been conducted to determine its real impact.

Based on patients analyzed, SARS-CoV-2 infects all age groups equally, except perhaps children and adolescents. One unanswered question is why some patients develop severe disease, while others do not. Among the different parameters that can influence the severity of this infection, we will focus on the role of the cellular immune response.

## Recent Progress in Immune Control of COVID-19 Pathogenesis

Usually, type I interferons (IFN-α/β) provide the first line of defense by generating cell-intrinsic antimicrobial states to limit virus replication. It seems, however, that pathogenic CoVs are particularly adapted to dampen responses mediated by IFN-α/β (25, 26). Several hypotheses were proposed to explain this early modulation of the immune response. It was shown that the Orf6 protein of SARS-CoV disrupts the karyopherin transport (27) and consequently inhibits the import of transcriptional factors, such as STAT1, into the nucleus, resulting in an inhibition of IFN response. Similarly, the Orf3b protein of SARS-CoV inhibits phosphorylation of interferon regulatory factor 3 (IRF3) (28), a protein involved in the activation of IFN-α/β. In China, the guidelines for the treatment of COVID-19 recommended administering IFN-α in combination with ribavirin (29), although no improvement was recorded. Interestingly, IFN-α effectively inhibited SARS-CoV replication but 50–90 times lower than IFN-β (30–32), suggesting that IFN-β could be a better antiviral component in patients' treatment. Thus, in the European DisCoVeRy trial, a combination of subcutaneous IFN-β with lopinavir/ritonavir is compared to hydroxychloroquine and remdesivir.

The loss of the “front line” antiviral defense mechanism mediated by IFN-α/β deficiency could be implicated in the induction of the cytokine storm leading to macrophage activation syndrome (MAS)-like pathology (33, 34). This cytokine storm is considered as the root cause of pathogenic inflammation in COVID-19. However, its initial trigger is not yet known, but it likely involves the immune system's detection of a large quantity of viral antigens released by dying cells. One in two fatal cases of COVID-19 experience a cytokine storm, 82% of whom are over the age of 60 (35). Interestingly, NLRP3, a major protein component of the inflammasome, could play a role. During aging, there is a steady increase in the abundance and activity of NLRP3 in immune cells in the lung, which contribute to pulmonary fibrosis (36). After age and hematological cancers, obesity is the next major risk factor for COVID-19 fatality, similar to type 2 diabetes. Obesity is well-known to increase the activity of NLRP3 and stimulate inflammation during viral infection (37).

**Figure 1:**
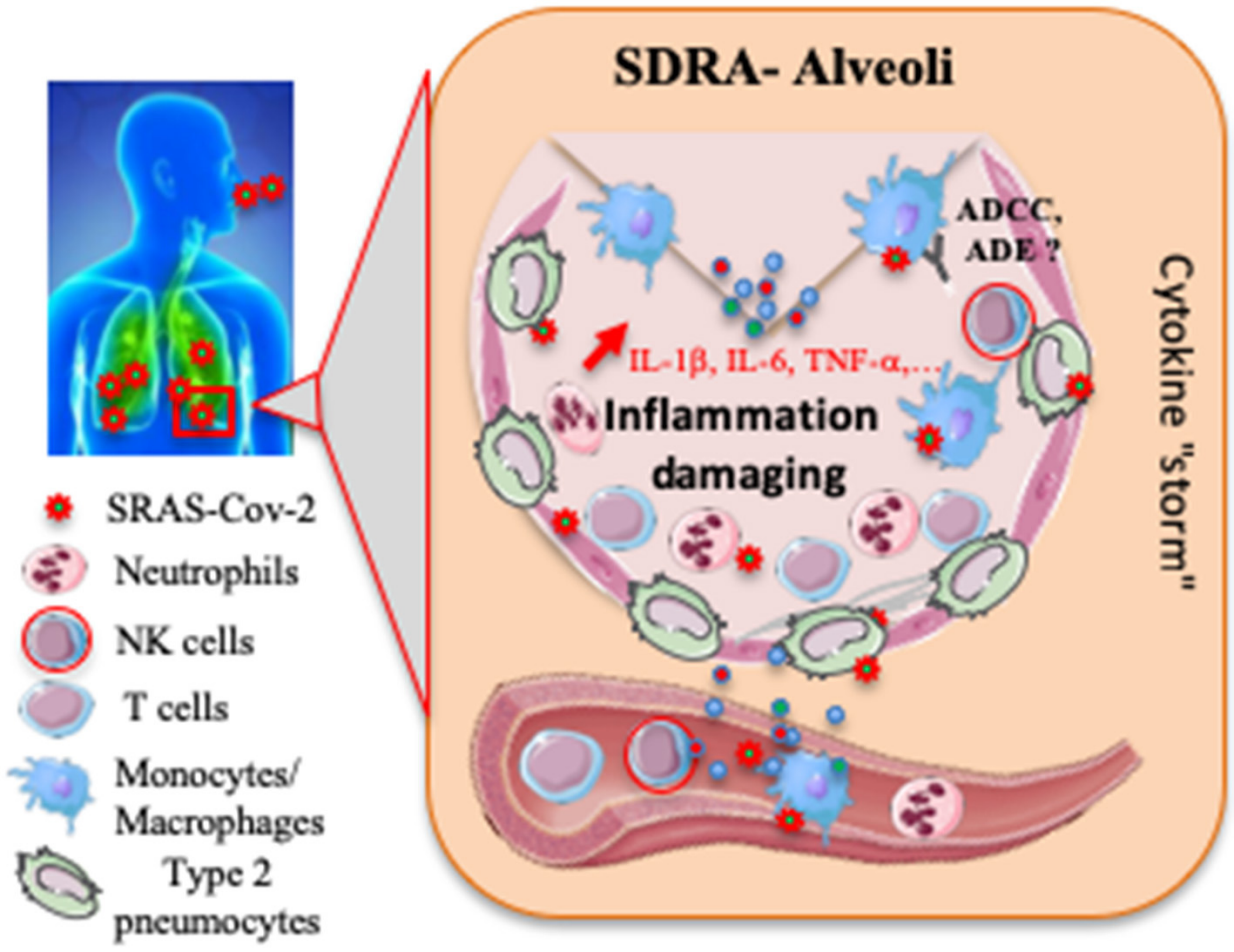
Proposed host immune responses during SARS-CoV-2 infection. Aerosolized uptake of SARS CoV-2 leads to infection of ACE2-expressing target cells, such as alveolar type 2 pneumocytes or other unknown target cells. The virus may dampen antiviral IFN-α/β responses resulting in uncontrolled viral replication. The influx of neutrophils and monocytes/macrophages results in hyperproduction of pro-inflammatory cytokines. The immunopathology of lung may be the result of the “cytokine storm.” NK cells and specific T cells may be activated and contribute to exacerbating inflammatory responses, and then to an acute respiratory distress syndrome (ARDS). SARS-CoV-2 specific Abs may help neutralize viruses, participate to antibody-dependent cell-mediated cytotoxicity (ADCC) or on the contrary to induce antibody-dependent enhancement (ADE). To date, most events remain speculative or unknown.

The cytokine storm is mainly associated with a high production of pro-inflammatory cytokines (i.e. IL-1β, IL-6, TNF-α) (Figure 1). For example, IL-6 production is about 3-fold higher in patients with complicated COVID-19 compared to asymptomatic patients (38). Preliminary data with tocilizumab, a humanized anti-IL-6 monoclonal antibody, in patients with COVID-19 pneumonia reveal clinical improvement in a small number of patients (39). Similarly, interferon gamma-induced protein 10 (IP-10) is correlated with patient viral load, whereas monocyte-chemotactic protein 3 (MCP3) is associated with loss of lung function (PaO_2_/FaO_2_ ratio), lung injury (Murray Score) and fatal outcome (40). Systemic inflammation was also observed in fatal cases of H1N1, with high IL-6 and IP-10 concentrations in the lungs, associated with massive infiltration of immune cells in the lung (41), also reported in severe or fatal forms of avian H5N1 and H7N9 pulmonary infection (42, 43).

### What of the Cell-Innate Immunity?

The epithelium of the lungs is the largest surface in the human body (>200 m^2^) in direct contact with the external environment. The lungs inhale daily about 10,000 l of air that contains various pathogenic particles, like the SARS-CoV-2 in fine droplets. Thus, this constant exposure to pathogens requires a very efficient immune system to sense the challenge and protect the host. To this end, the airways are endowed with physical barriers such as a layer of mucus, which is present over its entire surface to defend this tissue against pathogens, but also a vast network of cellular and humoral host defense mechanisms.

This network is mainly composed of epithelial cells of the respiratory tract, dendritic cells (DC) and alveolar macrophages. These cells trigger pro-inflammatory downstream immune responses in the presence of viral particles. Liao et al. (44) found that the depletion of tissue-resident alveolar macrophages and the accumulation of inflammatory macrophages in bronchoalveolar lavage cells were associated with disease severity. However, it would be necessary to finely test the infectivity of the monocyte/macrophage lineage with SARS-CoV-2 to determine better its impact on inflammatory responses. In this acute inflammatory reaction, neutrophils are also attracted and localized mainly in the bronchoalveolar space (45). Consistently, elevated neutrophil levels were reported in COVID-19^+^ patients (46, 47). Importantly, the lung constitutes the most important reservoir of neutrophils in the systemic circulation (~40% of total body neutrophils). It is plausible that elevated neutrophil level is associated with increased reactive oxygen species (ROS) and neutrophil extracellular traps (NETs), both considered as the most potent antimicrobial mechanisms used by neutrophils. Inappropriate levels of these neutrophil-derived products could contribute to the development of the “cytokine storm” initiated by the lung-infiltrating macrophages, and then to the partial destruction of lung tissues (Figure 1) (2, 48).

Mucosal-associated invariant T (MAIT) cells represent a population of innate T cells. They recognize metabolites that are presented by the major histocompatibility complex (MHC) class I-related protein MR1. Potential effectors of MAIT cell antimicrobial activity include the secretion of TNF-α, IFN-γ, IL-17A, and IL-22 as well as granzyme B and perforin (49, 50). Changes in MAIT cell frequencies have been reported in several viral infections; for example, higher cell numbers were found in survivors infected by H7N9 influenza, compared to samples from fatalities (51). Consistently, *in vitro* coculture of primary peripheral blood mononuclear cells and H7N9-infected A549 airway epithelial cells was associated with increased intracellular IFN-γ and granzyme B levels in MAIT cells (51). Very recent preliminary data also suggested a very significant decrease of MAIT cells in COVID-19^+^ patients; expression of the CD69 activation marker on blood MAIT cells at inclusion was predictive of COVID-19 severity (52).

Natural killer (NK) cells are another key element of innate immunity (53). It was rapidly determined that in COVID-19 patients, the total number of NK cells is markedly decreased (54), as previously reported for the SARS (55). NK cells express a variety of receptors that transduce either activating or inhibitory signals. Integration of these signals regulates the effector functions of NK cells, including cytotoxic activity and cytokine secretion (53, 56). In patients infected with SARS-CoV-2, NKG2A expression was significantly increased on NK cells (54). The CD94/NK group 2 member A (NKG2A) heterodimeric receptor is one of the most prominent NK inhibitory receptors. It binds to a non-classical minimally polymorphic HLA class I molecule (HLA-E), which presents peptides derived from leader peptide sequences of other HLA class I molecules (57). Upon ligation by peptide-loaded HLA-E, NKG2A transduces inhibitory signaling through 2 inhibitory immune-receptor tyrosine-based inhibition motifs, thus suppressing NK cytokine secretion and cytotoxicity (58). A clinical trial is ongoing in the presence of anti-NKG2A (Monalizumab) in Patients with advanced or metastatic cancer infected by SARS-CoV-2 (ClinicalTrials.gov Identifier: NCT04333914). However, more extensive phenotypic studies of NK cells will be necessary to determine the role of other cell markers and to measure their impact in disease evolution better. Consistent with increased NKG2A levels on NK cells from COVID-19 patients, low polyfunctional capacities were reported (54). Hence, SARS-CoV-2 may break down antiviral immunity mediated by NK cells at an early stage of infection, with putative consequences for the development of an efficient adaptive immunity. To increase NK-cell capability, a phase I clinical trial is ongoing to evaluate the safety and efficiency of allogenic NK-cell transfer in combination with standard therapy for 30 pneumonia patients infected with SARS-CoV-2 (ClinicalTrials.gov identifier: NCT04280224).

In other infectious situations, such as dengue virus infection, activation of NK cells by antibodies (Abs) can enhance controlled antibody-dependent enhancement (ADE) process (Figure 1), which occurs when Abs specific to a viral determinant facilitate secondary infection. Interestingly, it was shown previously that sera from SARS-CoV infected patients enhance viral entry into Fc receptor-expressing cells (59, 60). This mechanism should be extensively studied in a COVID-19 context to guide the development of future vaccine and antibody-based drug therapy.

Together, the preliminary data on COVID-19 patients suggest that SARS-CoV-2 could use different strategies to evade and/or antagonize different arms of the innate immune system.

### What of the Cell-Adaptive Immunity?

Severe lymphopenia was observed until death in non-survivor patients with COVID-19 (12). Consistently, the acute phase of SARS in human patients was associated with marked leukopenia in up to 80% of hospitalized patients, associated with a dramatic loss of CD4 and CD8 T cells (61, 62). In SARS-CoV-infected patients, it was shown that infection of T lymphocytes directly contributes to lymphopenia and atrophy of the spleen and lymphoid tissue (63). Lymphopenia is also observed in MERS patients, albeit to a lesser degree than in SARS patients (64). Understanding the mechanism of lymphopenia could open the way to the development of a new strategy for the treatment of COVID-19. Several potential mechanisms could be considered: (i) The virus might directly infect lymphocytes, resulting in lymphocyte death, as recently reported by Wang et al. (65) for the SARS-CoV-1. (ii) The virus can damage different target organs, such as bone marrow and thymus, which can no longer function normally. (iii) Inflammatory cytokines are massively produced, perhaps leading to lymphocyte apoptosis. (iv) Lymphocytes are trapped in infected tissues (Figure 1). Further research is needed to confirm these hypotheses. Importantly, the loss of lymphocytes was transient; CD8^+^ T lymphocytes and memory CD4^+^ T cells of SARS patients returned to normal within 2–3 and 12 months after infection, whereas other CD4^+^ T cell subsets were still lower than in healthy controls (66).

The first study on patients with COVID-19 revealed that low levels of IFN-γ and TNF-α in CD4^+^ T cells are associated with severity. Consistently, in CD8^+^ T cells, the frequency of the exhausted (PD-1^+^CTLA-4^+^TIGIT^+^) subset was significantly higher in the severe group (67). Consequently, the no (low) functionality of CD8^+^ T cells in severe patients could impact an efficient control of infection (67), as previously described in SARS-CoV infection (68). Furthermore, COVID-19 was associated with a significant decrease of T cell activation, determined by CD25, CD28, and CD69 expression on CD4^+^ and CD8^+^ T cell subsets (68). Despite a wave of information on the specific T cell responses to many other pathogens, less is known about respiratory CoV infections. CD8^+^ T cells are typically required for the control of influenza virus and other respiratory viruses (68). Furthermore, T resident memory cells (TRM) are critical in preventing re-infection from influenza virus (69). Their role in SARS-Co-V2 infection should be, however, more finely determined. In senescent mice infected by SARS-CoV, CD8^+^ CTLs alone are not sufficient to clear the virus in the absence of both CD4^+^ T cells and specific Abs (70).

On the other hand, depletion of CD4^+^ T cells in SARS-infected patients reduced production of neutralizing Abs and Th1 cytokines and induced lower recruitment of inflammatory monocytes in the lung. This mechanism can be bypassed by a passive transfer of neutralizing Abs against SARS-CoV, suggesting that the CD4-mediated control of infection most likely operates through Ab- and/or cytokine-dependent mechanisms. In fatal human fulminant cases of H1N1 influenza pneumonia that required mechanical ventilation, a strong effector T-cell response in the lungs was also observed in conjunction with high production of IFN-γ and IP-10, suggesting a massive and effective translocation of specific T cells to the lungs (41).

Genetic differences in HLA haplotypes are also key parameters, known to contribute to individual sensitivity against pathogens as previously described for tuberculosis, leprosy, HIV, hepatitis B, and influenza (71). For example, HLA-A^*^11, HLA-B^*^35, and HLA-DRB1^*^10 confer susceptibility to H1N1 infection (72). For SARS-CoV-2, a preliminary *in silico* analysis of viral peptide-MHC class-1 binding affinity suggests that individuals expressing HLA-B^*^46:01 may be particularly vulnerable to COVID-19, as previously shown for the SARS. At the same time, HLA-B^*^15:03 showed the greatest capacity to present highly conserved SARS-CoV-2 peptides shared among common human CoVs (73, 74). This observation suggests that the HLA distribution could affect the cellular immune response to SARS-CoV-2, and might explain the differences in COVID-19 susceptibility around the world. However, it seems crucial for the development of vaccine strategies to understand whether specific HLA haplotypes are associated with the development of anti-SARS-CoV-2 immunity. Interestingly, among the first 120 available SARS-CoV-2 sequences (as of February 21, 2020), several B cell and T cell epitopes specific to SARS-CoV-2 were identified for the spike and nucleocapsid proteins, that potentially induce protection against COVID-19 (75).

## Concluding Comments

Current observations indicate that SARS-CoV-2 is particularly adapted to evade immune responses at the early stage of infection. Most mechanisms are linked to inappropriate type 1 IFN responses, massive inflammatory cytokine production, and possibly to a defect in NK-cell functions. Preliminary data also suggest adaptive immune evasion, as indicated by the exhaustion of T lymphocytes. However, current evidence strongly indicated that the Th1-type response is key to the successful control of human pathogenic CoVs, in the association with the presence of specific neutralizing Abs. Although there are clear relationships between the severity of the disease and immune responses, the role of protective immunity currently remains questionable.

Alarmingly, some patients remain viral positive, while others even relapse, after discharge from hospital, as recently stated by WHO (3), suggesting that complete control of the virus by the immune response could be difficult to induce at least in some patients. This could also have an impact on the development of the second wave of the epidemic, which is currently strongly envisaged. The vaccine remains the best way to counter this epidemic. However, to define the surrogate parameters of vaccine efficacy, it should be important to better monitor T/B cell responses of recovered patients and to better understand the aging impact on the immune responses in COVID-19 patients, including the relative protection of younger individuals, excepted for some unexplained cases of Kawasaki-like syndrome. If overlapping epitopes among different human CoVs can be identified, this could help in the design of cross-reactive vaccines that protect against several pathogenic CoVs in the future.

## Data Availability Statement

All datasets presented in this study are included in the article.

## Author Contributions

All authors were involved in reading bibliography and writing the article. All co-authors reviewed the article.

## Conflict of Interest

The authors declare that the research was conducted in the absence of any commercial or financial relationships that could be construed as a potential conflict of interest.
